# Abnormal microRNA expression profile at early stages of gestation in pregnancies destined to develop placenta previa

**DOI:** 10.3389/fmed.2024.1469855

**Published:** 2024-12-03

**Authors:** Ilona Hromadnikova, Katerina Kotlabova, Ladislav Krofta

**Affiliations:** ^1^Department of Molecular Biology and Cell Pathology, Third Faculty of Medicine, Charles University, Prague, Czechia; ^2^Institute for the Care of the Mother and Child, Third Faculty of Medicine, Charles University, Prague, Czechia

**Keywords:** first trimester screening, gene expression, microRNAs, prediction, placenta previa, whole peripheral venous blood

## Abstract

**Background:**

Placenta previa is the abnormal implantation of the placenta into the lower segment of the uterus, is associated with adverse maternal and fetal outcomes such as placenta accreta spectrum disorders, antepartum and postpartum hemorrhage, fetal growth restriction, prematurity, stillbirth and neonatal death, thrombophlebitis, and septicemia. The aim of the study was to assess retrospectively how the later onset of placenta previa affects the microRNA expression profile in the whole peripheral blood during the first trimester of gestation.

**Methods:**

Regarding the occurrence of the association between aberrant microRNA expression profiles at early stages of gestation and later onset of various pregnancy-related complications, we selected for the study pregnancies developing placenta previa as the only pregnancy-related disorder. In total, 24 singleton pregnancies diagnosed with placenta previa that underwent first-trimester prenatal screening and delivered on-site within the period November 2012–May 2018 were included in the study. Overall, 80 normal pregnancies that delivered appropriate-for-gestational age newborns after completing 37 weeks of gestation were selected as the control group based on the equality of the length of biological sample storage.

**Results:**

Downregulation of multiple microRNAs (miR-20b-5p, miR-24-3p, miR-26a-5p, miR-92a-3p, miR-103a-3p, miR-130b-3p, miR-133a-3p, miR-145-5p, miR-146a-5p, miR-155-5p, miR-181a-5p, miR-195-5p, miR-210-3p, miR-342-3p, and miR-574-3p) was observed in pregnancies destined to develop placenta previa. The combination of seven microRNAs (miR-130b-3p, miR-145-5p, miR-155-5p, miR-181a-5p, miR-210-3p, miR-342-3p, and miR-574-3p) showed the highest accuracy (AUC 0.937, *p* < 0.001, 100.0% sensitivity, 83.75% specificity) to differentiate, at early stages of gestation, between pregnancies with a normal course of gestation and those with placenta previa diagnosed in the second half of pregnancy. Overall, 75% of pregnancies destined to develop placenta previa were correctly identified at 10.0% FPR.

**Conclusion:**

Consecutive large-scale analyses must be performed to verify the reliability of the proposed novel early predictive model for placenta previa occurring as the only pregnancy-related disorder.

## 1 Introduction

Placenta previa is diagnosed when the placenta obstructs the internal cervical os. There are three classification grades of placenta previa: marginal (placenta reaches the margin of the internal cervical os), partial (placenta partially covers the internal cervical os), and complete (placenta completely covers the internal cervical os) (1). The prevalence of placenta previa differs regionally but, overall, it reaches 5.2 cases per 1,000 pregnancies (2, 3). The pathogenesis of this placental disorder remains to be resolved. Placenta previa is associated with adverse maternal and fetal outcomes such as placenta accreta spectrum (PAS) disorders, antepartum and postpartum hemorrhage, fetal growth restriction (FGR), prematurity, stillbirth and neonatal death, thrombophlebitis, and septicemia (2, 4–7). Prior spontaneous or induced abortion, male fetus, smoking, advanced maternal age, C-section, and assisted reproductive techniques (singleton pregnancy) represent the main risk factors associated with placenta previa (8–10). The recommendations for diagnosis and classification of placenta previa and for managing the care of women have been summarized in the SOGC Clinical Practice Guideline (11). Screening for placenta previa is a part of the routine antenatal care performed at 18–22 gestational weeks and 32–34 gestational weeks (12). Currently, there is no screening protocol for placenta previa in the first trimester of gestation. However, recently, first-trimester screening for PAS disorders based on the early and late first-trimester sonographic markers suitable for individuals with a history of cesarean delivery has been introduced. A finding of the placenta under or within the scar niche should be referred to specialized centers (13).

MicroRNAs are small non-coding RNAs (18–25 nucleotides) that regulate gene expression at the post-transcriptional level (14, 15). MicroRNA upregulation results in the blockage of translation or degradation of mRNAs. On the other hand, microRNA downregulation results in overexpression of potential target genes. Usually, an altered microRNA expression profile accompanies certain diseases and may be used for the diagnosis and/or the assessment of prognosis (16–18).

The aim of the study was to assess if there are any changes in the microRNA expression profile in the whole peripheral blood in the first trimester of gestation in pregnancies developing placenta previa only in the second half of pregnancy. Recently, we observed an altered expression profile of microRNAs that play a role in the homeostasis and maintenance of the cardiovascular system and the pathophysiology of cardiovascular and cerebrovascular diseases in women at risk of adverse pregnancy outcomes (19–25). We have demonstrated the association between aberrant microRNA expression profiles at early stages of gestation and the presence of chronic hypertension (19), later onset of gestational hypertension (GH) (19), preeclampsia (PE) (19), FGR (20), small for gestational age (SGA) (20), preterm delivery (21), gestational diabetes mellitus (GDM) (22), HELLP syndrome (23), and stillbirth (24). Therefore, we intentionally excluded from the study pregnancies with placenta previa simultaneously affected with chronic hypertension and other pregnancy-related complications. We selected for the study pregnancies with placenta previa only (without the presence of abnormally invasive placenta such as placenta accreta, increta, or percreta).

## 2 Materials and methods

### 2.1 Patients cohort

The retrospective study in pregnancies of Caucasian descent was performed within the period 11/2012–5/2018. In total, 24 singleton pregnancies diagnosed with placenta previa in the second half of gestation, but with an otherwise normal course of gestation, were identified from a cohort of 3,028 pregnancies that underwent first-trimester prenatal screening and were delivered on-site. Only pregnancies without abnormally invasive placenta (placenta accreta, increta, or percreta) were included in the study. The reference group consisted of 80 normal pregnancies that delivered appropriate-for-gestational age newborns after completing 37 weeks of gestation. The selection of gestational-age-matched normal-term pregnancies at sampling (weeks) with equal biological sample-storage time ensured the homogeneity and comparability between the studied groups. A peripheral venous blood sampling was performed between 10 and 13 gestational weeks. Relevant clinical characteristics of patients are summarized in Table 1.

**Table 1:** Clinical characteristics of the control group and the group of complicated pregnancies.

	Normal pregnancies (*n* = 80)	Placenta previa (*n* = 24)	*p*-value OR 95% CI
Maternal characteristics			
Maternal age (years)	32 (25–42)	34 (23–42)	*p* = 0.005
Advanced maternal age (≥35 years old)	20 (25%)	11 (45.83%)	*p* = 0.054 OR: 2.538 0.983–6.558
Pre-pregnancy BMI (kg/m^2^)	21.28 (17.16–29.76)	21.04 (17.18–27.94)	*p* = 0.369
Diabetes mellitus (T1DM, T2DM)	0 (0%)	1 (4.17%)	–
Any autoimmune disease (SLE/APS/RA/MS/etc.)	0 (0%)	1 (4.17%) 1 MS	–
Chronic hypertension	0 (0%)	0 (0%)	-
Parity			
Nulliparous	40 (50.0%)	10 (41.67%)	*p* = 0.475 OR: 0.714 0.284–1.796
Parous	40 (50.0%)	14 (58.33%)	
Previous cesarean section (only parous women)			
0	34 (85.0%)	9 (64.29%)	*p* = 0.107 OR: 3.148 0.779–12.714
≥1	6 (15.0%)	5 (35.71%)	
Previous spontaneous abortion			
1	11 (13.75%)	4 (16.67%)	*p* = 0.406
2	4 (5.0%)	2 (8.33%)	
≥3	1 (1.25%)	2 (8.33%)	
Previous induced abortion	6 (7.50%)	6 (25.0%)	*p* = 0.026 OR: 4.111 1.186–14.254
Previous uterine surgery	17 (21.25%)	10 (41.67%)	*p* = 0.050 OR: 2.647 1.001–6.999
ART (IVF/ICSI/other)	2 (2.5%)	6 (25.0%)	*p* = 0.003 OR: 13.000 2.422–69.780
Smoking during pregnancy	2 (2.5%)	1 (4.17%)	*p* = 0.384 OR: 3.000 0.253–35.512
Pregnancy details (first trimester of gestation)			
Gestational age at sampling (weeks)	10.29 (9.57–13.71)	10.50 (9.71–13.71)	*p* = 0.173
Vaginal bleeding	0 (0%)	7 (29.17%)	*p* = 0.004 OR: 69.000 3.762–1265.43
Gestational age at delivery (weeks)	40.07 (37.57–42.0)	37.29 (32.00–39.86)	*p* < 0.001
Gestational age at delivery <37 weeks	0 (0%)	10 (41.67%)	–
Fetal birth weight (grams)	3,470 (2920–4,240)	3,035 (1440–4,050)	*p* < 0.001
Fetal sex			
Boy	40 (50.0%)	11 (45.83%)	*p* = 0.720 OR: 0.846 0.339–2.112
Girl	40 (50.0%)	13 (54.17%)	
Mode of delivery			
CS	11 (13.75%)	23 (95.83%)	*p* < 0.001 OR: 144.273 17.65–1179.15
Vaginal	69 (86.25%)	1 (4.17%)	

Continuous variables, compared using the Mann–Whitney test, are presented as median (range). Two categorical variables, presented as numbers (percent), were compared using the odds ratio test; three or more categorical variables were compared using the chi-squared test. *P*-value^:^ shows the comparison between normal pregnancies and pregnancies developing placenta previa. BMI, body mass index; T1DM, type 1 diabetes mellitus; T2DM, type 2 diabetes mellitus; SLE, systemic lupus erythematosus; APS, antiphospholipid syndrome; RA, rheumatoid arthritis; MS, multiple sclerosis; ART, assisted reproductive technology; IVF, *in vitro* fertilization; ICSI, intracytoplasmic sperm injection; CS, cesarean section.

All the included patients provided informed written consent for participation in the study. The Ethics Committee of the Third Faculty of Medicine, Charles University, granted initial approval for this study (implication of placental-specific microRNAs in maternal circulation for diagnosis and prediction of pregnancy-related complications, date of approval: 7 April 2011). Ongoing approval for the study was obtained from the Ethics Committee of the Third Faculty of Medicine, Charles University (long-term monitoring of complex cardiovascular profiles in mother, fetus, and offspring descending from pregnancy-related complications, date of approval: 27 March 2014) and the Ethics Committee of the Institute for the Care of the Mother and Child, Charles University (long-term monitoring of complex cardiovascular profiles in mother, fetus, and offspring descending from pregnancy-related complications, date of approval: 28 May 2015, number of approval: 1/4/2015). Informed consent is a complex process as it involves attaining consent for collecting peripheral blood samples at the beginning of pregnancy. In addition, it also includes gaining consent for collecting peripheral blood samples at the onset of pregnancy-related complications and collecting placental samples during childbirth in case of the onset of pregnancy-related complications.

### 2.2 Processing of samples and real-time RT-PCR analyses

Sample processing and real-time RT-PCR analysis were performed as previously described (19–25).

### 2.3 Statistical analysis

MicroRNA gene expression was compared between pregnancies with a normal course of gestation with and without the presence of placenta previa diagnosed in the second half of pregnancy using the Mann–Whitney test. The adjustment for covariates was performed using the Quade Non-parametric Analysis of Covariance (ANCOVA) (IBM SPSS Statistics 29.0.2.0). Benjamini–Hochberg-adjusted *p*-values were used to evaluate the statistical significance at *α* = 0.05 (*p* < 0.025*), *α* = 0.01 (*p* < 0.005**), and *α* = 0.001 (*p* < 0.001***) (26). Box plots were produced using the Statistica software (version 9.0; StatSoft, Inc., Tulsa, OK, United States).

Receiver operating characteristic (ROC) curves were produced using MedCalc software (MedCalc Software bvba, Ostend, Belgium). ROC curves displayed the areas under the curves (AUC) and the cutoff points associated with sensitivities, specificities, positive and negative likelihood ratios (LR+, LR−), and sensitivities at a 10.0% false-positive rate (FPR). To estimate the area under the curve (AUC) in the case of a combination of microRNA biomarkers, logistic regression was initially performed (MedCalc Software bvba, Ostend, Belgium). MicroRNAs entered into the initial logistic regression model as independent variables and the diagnosis as a dependent variable. Subsequently, a ROC curve analysis was performed (MedCalc Software bvba, Ostend, Belgium). The predictive probabilities gained from the logistic regression analysis were used as the new variable and the diagnosis as the classification variable.

### 2.4 Information on microRNA-gene-biological pathways interactions

The DIANA miRPath v.3 database (DIANA TOOLS-mirPath v.3^1^) and the genes union mode were used as an *a priori* analysis method to perform KEGG pathway enrichment analysis. This approach aimed to investigate the regulatory mechanisms of microRNAs dysregulated at the early stages of gestation in the whole peripheral blood of mothers destined to develop placenta previa. The TarBase v7.0 database, which contains experimentally verified microRNA targets, was preferentially used for this analysis. In case the TarBase v7.0 database did not provide a sufficient list of experimentally verified microRNA targets, the target prediction algorithm (microT-CDS v5.0) was used as an alternative. In addition, the search for interactions between microRNAs and genes associated with pathways (KEGG) was performed using miRWalk database v.3 and TargetScan, miRDB, and miRTarBase filters (miRWalk^2^).

## 3 Results

### 3.1 Altered expression profile of microRNAs during the first trimester of gestation in pregnancies with normal course of gestation that developed placenta previa in the second half of pregnancy

**Figure 1:**
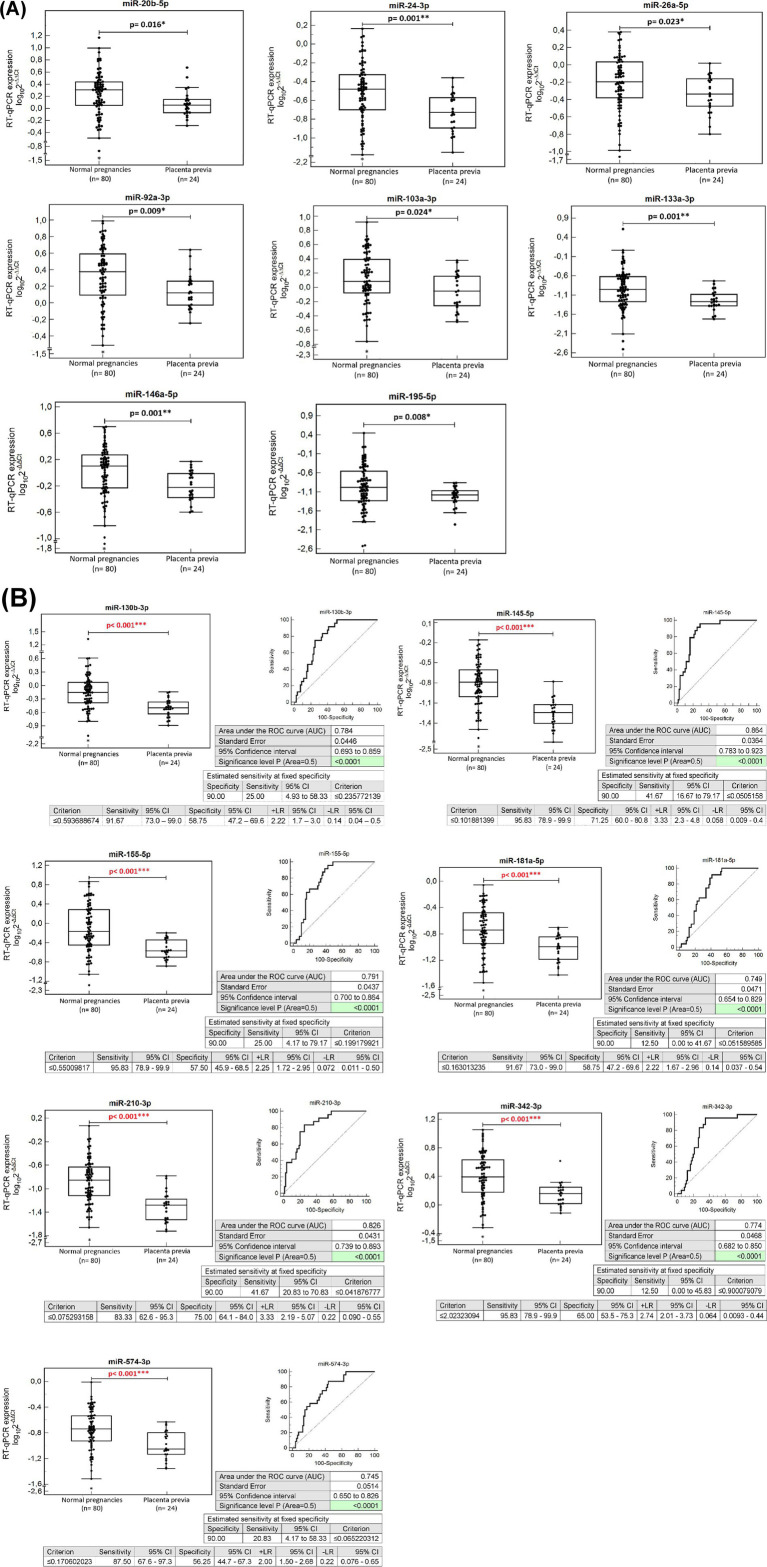
MicroRNA expression profile at early stages of gestation in pregnancies developing placenta previa. In total, 15 microRNAs differentiate between pregnancies with a normal course of gestation with and without the presence of placenta previa diagnosed in the second half of pregnancy. **(A)** Statistically significant microRNAs that were not used in the final microRNA combination. **(B)** Statistically significant microRNAs that were used in the final microRNA combination since they display excellent levels of differentiation using ROC analyses.

The expression profile of microRNAs was compared during the first trimester of gestation in whole peripheral blood samples between pregnancies with a normal course of gestation with and without the presence of placenta previa diagnosed in the second half of pregnancy. Downregulation of miR-20b-5p (*p* = 0.016*), miR-24-3p (*p* = 0.001**), miR-26a-5p (*p* = 0.023*), miR-92a-3p (*p* = 0.009*), miR-103a-3p (*p* = 0.024*), miR-130b-3p (*p* < 0.001***), miR-133a-3p (p = 0.001**), miR-145-5p (*p* < 0.001***), miR-146a-5p (p = 0.001**), miR-155-5p (p < 0.001***), miR-181a-5p (*p* < 0.001***), miR-195-5p (*p* = 0.008*), miR-210-3p (*p* < 0.001***), miR-342-3p (*p* < 0.001***), and miR-574-3p (*p* < 0.001***) was detected in pregnancies destined to develop placenta previa (Figure 1).

After adjustment for covariates (maternal age, pre-pregnancy BMI, previous cesarean section, previous uterine surgery, and ART), seven microRNAs remained statistically significant [miR-130b-3p (*p* = 0.009*), miR-145-5p (*p* < 0.001***), miR-155-5p (*p* = 0.006*), miR-181a-5p (*p* = 0.012*), miR-210-3p (*p* = 0.008*), miR-342-3p (*p* = 0.002**), and miR-574-3p (*p* = 0.005**)]. These seven microRNAs with excellent and acceptable levels of area under the curve (AUC) were able to differentiate between normal pregnancies with and without the presence of placenta previa with the following sensitivities at 10.0% false-positive rate (FPR) [miR-130b-3p (25.0%), miR-145-5p (41.67%), miR-155-5p (25.0%), miR-181a-5p (12.5%), miR-210-3p (41.67%), miR-342-3p (12.5%), and miR-574-3p (20.83%)] (Figure 1).

### 3.2 Combination of seven microRNA biomarkers differentiates at early stages of gestation between pregnancies with normal course of gestation with and without the presence of placenta previa diagnosed in the second half of pregnancy

**Figure 2:**
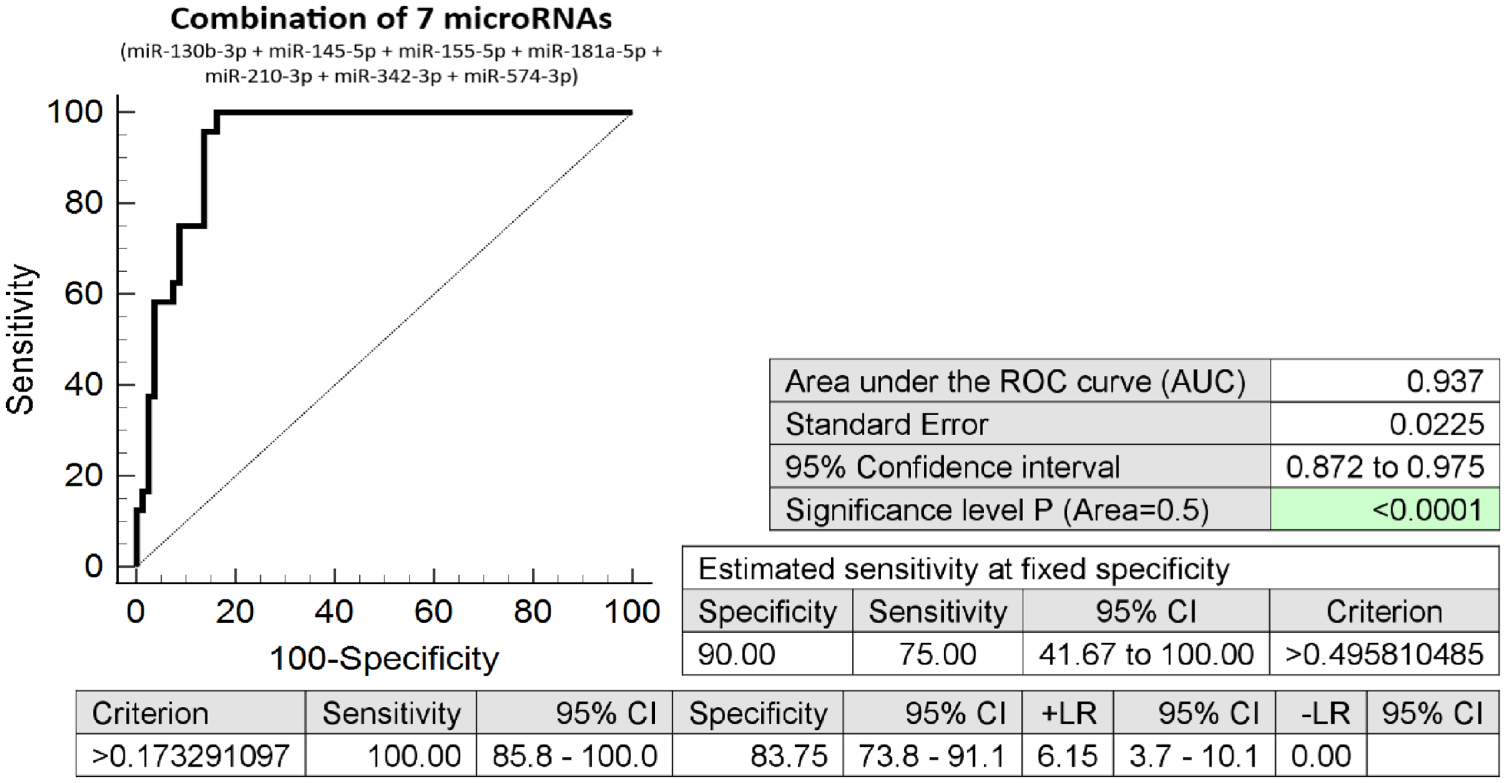
Combination of seven microRNA biomarkers – differentiation between pregnancies with and without the presence of placenta previa. The combination of 7 microRNA biomarkers (miR-130b-3p, miR-145-5p, miR-155-5p, miR-181a-5p, miR-210-3p, miR-342-3p, and miR-574-3p). Overall, 75% of pregnancies destined to develop placenta previa were revealed at early stages of gestation at 10.0% FPR.

The combination of seven microRNA biomarkers was able to differentiate, during the first trimester of gestation, between pregnancies with a normal course and those with placenta previa diagnosed in the second half of pregnancy, with very high accuracy (AUC 0.937, *p* < 0.001, 100.0% sensitivity, 83.75% specificity, and cutoff >0.17329). Overall, 75% of pregnancies destined to develop placenta previa were identified at 10.0% FPR (Figure 2).

### 3.3 Information on microRNA-gene-biological pathways interactions

The microRNA/gene/KEGG pathway analyses revealed the involvement of particular microRNAs with altered expression in various biological pathways and processes involved in placental development and maintenance of placental homeostasis (Table 2).

**Table 2:** Involvement of microRNAs in biological pathways and processes involved in placental development and maintenance of placental homeostasis.

miRNA	KEGG pathway database	Target gene	Biological process	Source for miRNA-target data
miR-130b-3p	TGF-beta signaling pathway	SMAD4	Angiogenesis	TarBase
		ACVR1	Placenta formation	TarBase
		INHBA		
		SMAD4		
	FoxO signaling pathway	BCL2L11	Apoptosis	TarBase
miR-145-5p	TGF-beta signaling pathway	SMAD2	Angiogenesis	TarBase
		SMAD4		
		SMAD2	Apoptosis	TarBase
		SMAD4		
		ACVR1	Placenta formation	TarBase
		SMAD2		
		SMAD4		
	PI3K-AKT signaling pathway	AKT1	Apoptosis	TarBase
	Thyroid hormone signaling pathway	CCND1	Angiogenesis	TarBase
		RCAN1		
		MYC		
miR-155-5p	TGF-beta signaling pathway	SMAD3	Angiogenesis	TarBase
		SMAD4		
		SMAD3	Apoptosis	TarBase
		SMAD4		
		ACVR2A	Placenta formation	TarBase
		SMAD3		
		SMAD4		
miR-181a-5p	TGF-beta signaling pathway	ACVR2A	Placenta formation	TargetScan
	HIF-1 signaling pathway	ANGPT2	Angiogenesis	TarBase
		FLT1		
		SERPINE1		
	PI3K-AKT signaling pathway	BCL2	Apoptosis	TarBase
miR-210-3p	TGF-beta signaling pathway	NCOR1	Apoptosis	miRDB
		NCOR1	Angiogenesis	
miR-342-3p	Thyroid hormone signaling pathway	CCND1	Angiogenesis	TarBase
		RCAN1		
	TGF-beta signaling pathway	SMAD2	Angiogenesis	TarBase
		SMAD2	Placenta formation	TarBase
miR-574-3p	TGF-beta signaling pathway	SMAD4	Angiogenesis	miRTarBase
		SMAD4	Apoptosis	miRTarBase
		SMAD4	Placenta formation	miRTarBase

SMAD2/3/4, small mother against decapentaplegic homolog 2/3/4; ACVR1, activin A receptor type 1; INHBA, inhibin subunit beta A; BCL2L11, Bcl2-like protein 11; AKT1, AKT serine/threonine kinase 1; CCND1, cyclin D1; RCAN1, regulator of calcineurin 1; MYC, MYC proto-oncogene, bHLH transcription factor; ACVR2A, activin A receptor type 2A; ANGPT2, angiopoietin 2; FLT1, fms-related receptor tyrosine kinase 1; SERPINE1, serpin family E member 1; BCL2, BCL2 apoptosis regulator; NCOR1, nuclear receptor corepressor 1.

## 4 Discussion

At early stages of gestation, we detected decreased expression of multiple microRNAs (miR-20b-5p, miR-24-3p, miR-26a-5p, miR-92a-3p, miR-103a-3p, miR-130b-3p, miR-133a-3p, miR-145-5p, miR-146a-5p, miR-155-5p, miR-181a-5p, miR-195-5p, miR-210-3p, miR-342-3p, and miR-574-3p) in pregnancies that developed placenta previa in the second half of pregnancy as the only pregnancy-related disorder. MicroRNA expression profiles associated with placenta previa resembled microRNA expression profiles associated with preterm birth [spontaneous preterm birth (PTB) or preterm prelabor rupture of membranes (PPROM)] in the absence of other pregnancy-related complications (21), where some of these microRNAs (miR-20b-5p, miR-24-3p, miR-26a-5p, miR-92a-3p, miR-133a-3p, miR-145-5p, miR-146a-5p, miR-155-5p, miR-210-3p, and miR-342-3p) were also downregulated at the early stages of gestation. The microRNA expression profile associated with placenta previa was identical to the microRNA expression profile associated with late miscarriage or stillbirth (24), where microRNAs such as miR-130b-3p, miR-145-5p, miR-210-3p, miR-342-3p, and miR-574-3p were also observed to have decreased expression at the early stages of gestation. The combination of seven microRNAs (miR-130b-3p, miR-145-5p, miR-155-5p, miR-181a-5p, miR-210-3p, miR-342-3p, and miR-574-3p) showed the highest accuracy (AUC 0.937, *p* < 0.001, 100.0% sensitivity, 83.75% specificity) to differentiate, at early stages of gestation, between pregnancies with normal course of gestation and those with placenta previa diagnosed in the second half of pregnancy. Overall, 75% of pregnancies destined to develop placenta previa were correctly identified at 10.0% FPR.

To the best of our knowledge, no study on the microRNA expression profile in the whole peripheral blood at the first trimester of gestation in pregnancies destined to develop placenta previa as the only pregnancy-related complication is available. Only a few studies examined microRNA expression profiles in pregnancies with placenta previa or abnormally invasive placenta (placenta accreta, increta, and percreta) in various biological samples (mainly in plasma or serum) at various stages of gestation (mainly at the third trimester of gestation) with the aim to predict these placental disorders. Hasegawa et al. reported that plasma miR-517a may serve at 32 weeks of gestation in pregnancies with placenta previa as a predictive marker for the risk of alert bleeding and massive hemorrhage at the delivery (27). Timofeeva et al. (28) observed abnormal plasma levels of miR-17-5p, miR-21-5p, miR-25-3p, miR-92a-3p, and miR-320a-3p at 30–34 gestational weeks in pregnancies with placenta accreta, increta, or percreta. The study by Munoz et al. (29) showed that plasma exosomal microRNAs (miR-92, −103, and −192) may represent additional potential biomarkers for detecting PAS within 25–36 gestational weeks. Similarly, Chen et al. (30) validated four other serum microRNAs (miR-139-3p, miR-196a-5p, miR-518a-3p, and miR-671-3p) that could be potentially used for non-invasive prenatal PAS screening during the third trimester and before the delivery. None of these stated microRNA biomarkers are identical to those we demonstrated to be dysregulated at early stages of gestation in pregnancies affected with placenta previa with otherwise normally ongoing gestation.

Consecutive large-scale analyses must be performed to verify the reliability of the proposed novel early predictive model for placenta previa occurring as the only pregnancy-related disorder based on the combination of microRNA biomarkers (miR-130b-3p, miR-145-5p, miR-155-5p, miR-181a-5p, miR-210-3p, miR-342-3p, and miR-574-3p). Multicenter studies will be needed to acquire a sufficient number of novel cases to validate the data resulting from the current pilot study. In addition, future studies tracking microRNAs with abnormal expression at early stages of gestation throughout the pregnancy are needed as well. If satisfactory discrimination power is achieved, gynecologists and obstetricians could have at their disposal a feasible, cost-effective way of identifying pregnancies at risk of placenta previa at early gestational stages when it occurs as the only pregnancy-related disorder.

## Data Availability

The raw data supporting the conclusions of this article will be made available by the authors, without undue reservation.

## Footnotes

^1^ https://www.uth.gr

^2^ https://www.uni-heidelberg.de/en

## References

[1] Bi S Zhang L Wang Z Chen J Tang J Gong J. Effect of types of placenta previa on maternal and neonatal outcomes: a 10-year retrospective cohort study. Arch Gynecol Obstet. (2021) 304:65–72. doi: 10.1007/s00404-020-05912-9, PMID: 33386958

[2] Sahu SA Shrivastava D. Maternal and perinatal outcomes in placenta Previa: a comprehensive review of evidence. Cureus. (2024) 16:e59737. doi: 10.7759/cureus.59737, PMID: 38841031 PMC11151188

[3] Jansen CHJR Kastelein AW Kleinrouweler CE Van Leeuwen E De Jong KH Pajkrt E. Development of placental abnormalities in location and anatomy. Acta Obstet Gynecol Scand. (2020) 99:983–93. doi: 10.1111/aogs.13834, PMID: 32108320 PMC7496588

[4] Karami M Jenabi E Fereidooni B. The association of placenta previa and assisted reproductive techniques: a meta-analysis. J Matern Fetal Neonatal Med. (2018) 31:1940–7. doi: 10.1080/14767058.2017.1332035, PMID: 28514884

[5] Hessami K Mitts M Zargarzadeh N Jamali M Berghella V Shamshirsaz AA. Ultrasonographic cervical length assessment in pregnancies with placenta previa and risk of perinatal adverse outcomes: a systematic review and meta-analysis. Am J Obstet Gynecol MFM. (2024) 6:101172. doi: 10.1016/j.ajogmf.2023.101172, PMID: 37778698

[6] Hu H Wang L Gao J Chen Z Chen X Tang P. Risk factors of severe postpartum hemorrhage in pregnant women with placenta previa or low-lying placenta: a retrospective cohort study. BMC Pregnancy Childbirth. (2024) 24:674. doi: 10.1186/s12884-024-06876-3, PMID: 39407169 PMC11475889

[7] Kumari U Naniwal A Rani V Chandat R Yadav S Pipal DK. A study of clinical characteristics, demographic characteristics, and fetomaternal outcomes in cases of placenta Previa: an experience of a tertiary care center. Cureus. (2022) 14:e32125. doi: 10.7759/cureus.32125, PMID: 36601148 PMC9805694

[8] Jenabi E Salimi Z Bashirian S Khazaei S Ayubi E. The risk factors associated with placenta previa: an umbrella review. Placenta. (2022) 117:21–7. doi: 10.1016/j.placenta.2021.10.009, PMID: 34768164

[9] Pun I Singh A. Feto-maternal outcomes in placenta Previa with and without previous cesarean section. J Nepal Health Res Counc. (2022) 20:142–6. doi: 10.33314/jnhrc.v20i01.3640, PMID: 35945867

[10] Post RJ Chang J Ziogas A Crosland BA Silver RM Haas DM. Risk factors and perinatal outcomes for persistent placenta previa in nulliparas. Am J Obstet Gynecol MFM. (2023) 5:101136. doi: 10.1016/j.ajogmf.2023.101136, PMID: 37598887

[11] Jain V Bos H Bujold E. Guideline No. 402: diagnosis and Management of Placenta Previa. J Obstet Gynaecol Can. (2020) 42:906–917.e1. doi: 10.1016/j.jogc.2019.07.019, PMID: 32591150

[12] Bhide A. Routine screening for placenta accreta spectrum. Best Pract Res Clin Obstet Gynaecol. (2023) 90:102392. doi: 10.1016/j.bpobgyn.2023.102392, PMID: 37541113

[13] Dar P Doulaveris G. First-trimester screening for placenta accreta spectrum. Am J Obstet Gynecol MFM. (2024) 6:101329. doi: 10.1016/j.ajogmf.2024.101329, PMID: 38447672

[14] Lai EC. Micro RNAs are complementary to 3' UTR sequence motifs that mediate negative post-transcriptional regulation. Nat Genet. (2002) 30:363–4. doi: 10.1038/ng865, PMID: 11896390

[15] Bartel DP. MicroRNAs: genomics, biogenesis, mechanism, and function. Cell. (2004) 116:281–97. doi: 10.1016/s0092-8674(04)00045-5 14744438

[16] Piletič K Kunej T. MicroRNA epigenetic signatures in human disease. Arch Toxicol. (2016) 90:2405–19. doi: 10.1007/s00204-016-1815-7, PMID: 27557899

[17] Wang J Chen J Sen S. MicroRNA as biomarkers and diagnostics. J Cell Physiol. (2016) 231:25–30. doi: 10.1002/jcp.25056, PMID: 26031493 PMC8776330

[18] Condrat CE Thompson DC Barbu MG Bugnar OL Boboc A Cretoiu D. miRNAs as biomarkers in disease: latest findings regarding their role in diagnosis and prognosis. Cells. (2020) 9:276. doi: 10.3390/cells9020276, PMID: 31979244 PMC7072450

[19] Hromadnikova I Kotlabova K Krofta L. Cardiovascular disease-associated MicroRNA dysregulation during the first trimester of gestation in women with chronic hypertension and normotensive women subsequently developing gestational hypertension or preeclampsia with or without fetal growth restriction. Biomedicines. (2022) 10:256. doi: 10.3390/biomedicines10020256, PMID: 35203467 PMC8869238

[20] Hromadnikova I Kotlabova K Krofta L. First-trimester screening for fetal growth restriction and small-for-gestational-age pregnancies without preeclampsia using cardiovascular disease-associated MicroRNA biomarkers. Biomedicines. (2022) 10:718. doi: 10.3390/biomedicines10030718, PMID: 35327520 PMC8945808

[21] Hromadnikova I Kotlabova K Krofta L. First trimester prediction of preterm delivery in the absence of other pregnancy-related complications using cardiovascular-disease associated MicroRNA biomarkers. Int J Mol Sci. (2022) 23:3951. doi: 10.3390/ijms23073951, PMID: 35409311 PMC8999783

[22] Hromadnikova I Kotlabova K Krofta L. Cardiovascular disease-associated MicroRNAs as novel biomarkers of first-trimester screening for gestational diabetes mellitus in the absence of other pregnancy-related complications. Int J Mol Sci. (2022) 23:10635. doi: 10.3390/ijms231810635, PMID: 36142536 PMC9501303

[23] Hromadnikova I Kotlabova K Krofta L. First-trimester screening for HELLP syndrome-prediction model based on MicroRNA biomarkers and maternal clinical characteristics. Int J Mol Sci. (2023) 24:5177. doi: 10.3390/ijms24065177, PMID: 36982251 PMC10049724

[24] Hromadnikova I Kotlabova K Krofta L. First-trimester screening for miscarriage or stillbirth-prediction model based on MicroRNA biomarkers. Int J Mol Sci. (2023) 24:10137. doi: 10.3390/ijms241210137, PMID: 37373283 PMC10299132

[25] Hromadnikova I Kotlabova K Krofta L. First-trimester predictive models for adverse pregnancy outcomes—a base for implementation of strategies to prevent cardiovascular disease development. Front Cell Dev Biol. (2024) 12:1461547. doi: 10.3389/fcell.2024.1461547, PMID: 39296937 PMC11409004

[26] Haynes W. Benjamini–Hochberg Method In: Dubitzky W Wolkenhauer O Cho KH Yokota H, editors. Encyclopedia of systems biology. New York, NY: Springer (2013)

[27] Hasegawa Y Miura K Higashijima A Abe S Miura S Yoshiura K. Increased levels of cell-free miR-517a and decreased levels of cell-free miR-518b in maternal plasma samples from placenta Previa pregnancies at 32 weeks of gestation. Reprod Sci. (2015) 22:1569–76. doi: 10.1177/1933719115589407, PMID: 26040940

[28] Timofeeva AV Fedorov IS Pirogova MM Vasilchenko ON Chagovets VV Ezhova LS. Clusterin and its potential regulatory microRNAs as a part of Secretome for the diagnosis of abnormally invasive placenta: Accreta, increta, and Percreta cases. Life. (2021) 11:270. doi: 10.3390/life11040270, PMID: 33805203 PMC8064394

[29] Munoz JL Einerson BD Silver RM Mulampurath S Sherman LS Rameshwar P. Serum exosomal microRNA pathway activation in placenta accreta spectrum: pathophysiology and detection. AJOG Glob Rep. (2024) 4:100319. doi: 10.1016/j.xagr.2024.100319, PMID: 38440154 PMC10910333

[30] Chen S Pang D Li Y Zhou J Liu Y Yang S. Serum microRNA biomarker discovery for placenta accreta spectrum. Placenta. (2020) 101:215–20. doi: 10.1016/j.placenta.2020.09.068, PMID: 33017714

